# Supplementary material for: Evaluation of the 22G Franseen needle and 22G Lancet needle for endoscopic ultrasonography-guided tissue acquisition sampling in solid pancreatic lesions: Propensity score weighting
Source: PLoS One. 2025 May 16;20(5):e0322857. doi: 10.1371/journal.pone.0322857 (PMC12084063; doi:10.1371/journal.pone.0322857)
Supplement: S1 Table — (DOCX) [file pone.0322857.s002.docx]

| S 1 Table. Diagnostic abilities of a Lancet and Franseen needle. | | | |
| --- | --- | --- | --- |
|  |  |  |  |
|  | **Lancet needle (n = 174)** | **Franseen needle (n = 98)** | **p value** |
| ***Cytology*** |  |  |  |
| Sensitivity, % | 77.2 (0.69–0.84) | 83.5 (0.74–0.91) | 0.284 |
| Specificity, % | 97.9 (0.89–1.00) | 100 (0.93–1.00) | 0.497 |
| PPV^‡^, % | 99.0 (0.95–1.00) | 100 (0.93–1.00) | 1.000 |
| NPV^§^, % | 61.3 (0.50–0.72) | 48.1 (0.29–0.68) | 0.089 |
| Diagnostic accuracy, % | 82.8 (0.76–0.88) | 85.7 (0.77–0.92) | 0.696 |
| ***Combined (Cytology and Histology)*** | |  |  |
| Sensitivity, % | 81.1 (0.73–0.88) | 91.8 (0.84–0.97) | 0.037 |
| Specificity, % | 100 (0.89–1.00) | 100 (0.66–1.00) | 1.000 |
| PPV^‡^, % | 100 (0.95–1.00) | 100 (0.93–1.00) | 1.000 |
| NPV^§^, % | 66.2 (0.80–0.91) | 65.0 (0.41–0.85) | 1.000 |
| Diagnostic accuracy, % | 86.2 (0.80–0.91) | 92.9 (0.86–0.97) | 0.165 |
| §: NPV, Negative predictive value; ‡: PPV, Positive predictive value. | | |  |

| S 2. The diagnostic performance for malignant/benign lesions according to the needle type. | | | |  |
| --- | --- | --- | --- | --- |
|  |  |  |  |  |
|  | **Lancet needle** | **Franseen needle** |  | **p value** |
| Cytological diagnostic accuracy for benign, % | 97.9 (0.89-1.00) | 100 (0.66-1.00) |  | 0.497 |
| Histological diagnostic accuracy for benign, % | 100 (0.89-1.00) | 100 (0.66-1.00) |  | 1.000 |
| Cytological and Histological diagnostic accuracy for benign, % | 100 (0.89-1.00) | 100 (0.66-1.00) |  | 1.000 |
| Cytological diagnostic accuracy for malignancy, % | 77.0 (0.69-0.84) | 83.5 (0.74-0.91) |  | 0.093 |
| Histological diagnostic accuracy for malignancy, % | 59.8 (0.51-0.68) | 82.4 (0.73-0.90) |  | <0.001 |
| Cytological and Histological diagnostic accuracy for malignancy, % | 81.1 (0.73-0.88) | 91.8 (0.84-0.97) |  | 0.046 |
