# Supplementary material for: Evaluation of the 22G Franseen needle and 22G Lancet needle for endoscopic ultrasonography-guided tissue acquisition sampling in solid pancreatic lesions: Propensity score weighting
Source: PLoS One. 2025 May 16;20(5):e0322857. doi: 10.1371/journal.pone.0322857 (PMC12084063; doi:10.1371/journal.pone.0322857)
Supplement: S2 Table — (DOCX) [file pone.0322857.s003.docx]

| S 2 Table. The diagnostic performance for malignant/benign lesions according to the needle type. | | | |  |
| --- | --- | --- | --- | --- |
|  |  |  |  |  |
|  | **Lancet needle** | **Franseen needle** |  | **p value** |
| Cytological diagnostic accuracy for benign, % | 97.9 (0.89–1.00) | 100 (0.66–1.00) |  | 0.497 |
| Histological diagnostic accuracy for benign, % | 100 (0.89–1.00) | 100 (0.66–1.00) |  | 1.000 |
| Cytological and Histological diagnostic accuracy for benign, % | 100 (0.89–1.00) | 100 (0.66–1.00) |  | 1.000 |
| Cytological diagnostic accuracy for malignancy, % | 77.0 (0.69–0.84) | 83.5 (0.74–0.91) |  | 0.093 |
| Histological diagnostic accuracy for malignancy, % | 59.8 (0.51–0.68) | 82.4 (0.73–0.90) |  | <0.001 |
| Cytological and Histological diagnostic accuracy for malignancy, % | 81.1 (0.73–0.88) | 91.8 (0.84–0.97) |  | 0.046 |
