# Supplementary material for: Evaluation of the 22G Franseen needle and 22G Lancet needle for endoscopic ultrasonography-guided tissue acquisition sampling in solid pancreatic lesions: Propensity score weighting
Source: PLoS One. 2025 May 16;20(5):e0322857. doi: 10.1371/journal.pone.0322857 (PMC12084063; doi:10.1371/journal.pone.0322857)
Supplement: Attachment — Submitted filename: PONE COMMENTS.docx [file pone.0322857.s004.docx]

In this original article entitled “Evaluation of the 22G Franseen needle and 22G Lancet needle for EUS-FNAB sampling in solid pancreatic lesions: Propensity score weighting,” Yuki Ishikawa-Kakiya et al. conducted a retrospective comparative study aimed to evaluate the histologic accuracy of two needles in pancreatic lesions.

The study is well written, with a clear and concise presentation and detailed method section. The use of a single technique and the relatively low number of patients included are limitations of the study. However, using a propensity score has mitigated the bias, and the results align with the literature. I have some suggestions:

-In the section “true and false diagnosis” the inadequate sample definition is confounding (line 143). Not adequate and not accurate are different definitions, as correctly signed in the following article paragraphs.

-in the discussion the impact of the size of the lesion and the benign nature (especially Autoimmune pancreatitis) should be cited.

-please correct the typo Franssen (line 308).

The article may be accepted after minor revisions.
