# Supplementary material for: Evaluation of the 22G Franseen needle and 22G Lancet needle for endoscopic ultrasonography-guided tissue acquisition sampling in solid pancreatic lesions: Propensity score weighting
Source: PLoS One. 2025 May 16;20(5):e0322857. doi: 10.1371/journal.pone.0322857 (PMC12084063; doi:10.1371/journal.pone.0322857)
Supplement: Attachment — Submitted filename: PLOSONE response final.docx [file pone.0322857.s006.docx]

**Point-by-Point Responses (**PONE-D-32776**)**

**REVIEWER'S COMMENTS TO THE AUTHOR:**

**Reviewer #1**

**Comment 1:**

The article is interesting although this topic should be addressed in a RCT to obviate to the risk of selection bias.

**Responses to the Reviewer’s comments:**

Thank you for your insightful comments. We completely agree that a randomized controlled trial (RCT) would be the best approach, and some studies have already reported before. In those studies, the accuracy of Franseen needle was higher than traditional FNA needle [1-3]. However, tissue obtained from EUS-FNAB is traditionally evaluated using cytology and histology, which has not been discussed about the details of diagnosis method; cytological, histological or combined accuracy is limited in the Franseen needle and Lancet needle. In this study, we compared various diagnostic methods, with a particular focus on histological diagnosis. In addition, we examined the amount of blood contamination, effect of which has not been sufficiently evaluated in previous studies.

This comparison was conducted through a retrospective observational study in order to increase the sample size. We acknowledge the potential issue of selection bias inherent in retrospective studies. To address this, we used propensity score weighting with the IPTW method to minimize bias and make the analysis more akin to a pseudo-RCT. We hope this approach is satisfactory and meets your expectations.

**Comment 2:**
I am impressed with the low accuracy and sensitivity in this series, particularly with lancet needles. This is not in keeping with the current literature and with the experience of this reviewer and of the scientific community in general.

**Responses to the Reviewer’s comments:**

Thank you for your insightful comments. We acknowledge that the diagnostic accuracy and sensitivity of the lancet needle in our study are lower than those reported in recent literature and may not align with the broader experience of the scientific community.

However, some previous reports have also shown a lower diagnostic accuracy for the lancet needle, with rates as low as 63.5%[4]. In our study, this lower accuracy may be partially attributed to the inclusion of earlier cases of EUS-FNA performed at our institution, when both endoscopic technique and cytological evaluation were still in their developmental stages. This learning curve effect likely impacted the overall diagnostic performance of the lancet needle.

Another important factor may be the amount of sample obtained. The amount of tissue collected with the lancet needle was often small. Small amounts of tissue increase the possibility of histological misinterpretation, especially in the presence of inflammation, which may mask or mimic pathological findings and reduce diagnostic accuracy. To examine this issue, we performed a sub-analysis limited to cases from 2016 onwards (n = 196), when the Franceen needle became available. The diagnostic accuracy of the Lancet needle was 64.3%, and that of the Franseen needle was 85.8%. The diagnostic accuracy of both the Lancet needle and Franseen needle has increased, which may have improved the skills of the technologists and endoscopists. In addition, univariate and multivariate analyses were performed on cases from 2016 onwards, and the tissue diagnostic rate of the FNB needle was shown to be significantly better.

|  | Univariate | |  | Multivariate | |
| --- | --- | --- | --- | --- | --- |
|  | Crude OR (95% CI^\|^) | p value |  | Crude OR (95% CI) | p value |
| **Needle, Franseen** | 2.88 (1.43–5.82) | 0.003 |  | 2.42 (1.17-5.02) | 0.018 |
| **Number of punctures** | 0.63 (0.45–0.89) | 0.008 |  | 0.71 (0.50-1.17) | 0.056 |

**Comment 3:**

The authors should perform several subgroup analyses based on some technical factors, for example the use of suction. These aspects should be commented in the discussion (cite the recent papers PMID: 36657607 and PMID: 35915956 )

The authors should consider the above reported technical features in the covariates used to build the propensity score model

**Responses to the Reviewer’s comments:**

Thank you for your valuable suggestions. I agree that technical factors such as aspiration technique may affect diagnostic results. Recent studies have demonstrated that wet-suction, both with FNB and FNA needles, can provide better tissue integrity, higher cell density, and better diagnostic accuracy compared with standard aspiration and slow pull techniques[5] [6]. These findings highlight the potential impact of the suction method on EUS-FNAB performance. However, as our study is retrospective in nature, detailed information regarding the aspiration method (e.g., wet suction or suction) was not consistently recorded across all cases. This lack of data precluded us from conducting subgroup analyses based on these technical factors. We acknowledge this as a limitation of our study and have clearly addressed it in the revised manuscript (page 30, line 403-409), “**First, we could not evaluate the relationship between factors such as the utilization of suction method (general suction or wet-suction), however, these factors may potentially influence diagnostic accuracy. Previous reports have indicated that wet suction resulted in blood contamination similar to that of the general suction method, but yielded better results in terms of diagnostic rate and tissue quality. Similarly, the puncture technique such as door knocking method or fanning method may affect diagnostic accuracy; however, due to insufficient data on this factor, it could not be fully evaluated in this study.**”. We greatly appreciate the reviewer’s insightful comments, which will guide future studies. Moving forward, prospective studies with standardized documentation of technical variables, including suction methods, are warranted to better elucidate their impact on diagnostic outcomes.

**Comment 4:**

Several different needles were included under the umbrella definition of lancet needle. The authors should specify some subgroup analysis based on the exact needle design

**Responses to the Reviewer’s comments:**

Thank you very much for your valuable comments. As you mentioned, the sono tip and expect are lancet needles, while the EZshot3plus is a Menghini needle [7]. However, all of these are considered FNA needles, and it has been reported that the EZ shot3 plus is included in the lancet needle group [8]. The Menghini needle is said to have a sharper lancet tip, which improves puncture performance. Therefore, I believe there is no issue with the classification. Furthermore, if we were to conduct subgroup analysis comparing lancet needles and Menghini needles, the sample size would be very small, making the results less reliable. However, we will consider adding further clarification or subgroup analysis if necessary in the revised manuscript.

**Comment 5:**

Any data on the adequacy for molecular analysis? IHC?

**Responses to the Reviewer’s comments:**

Thank you for your comments. Unfortunately, in our institution, molecular analysis and immunohistochemistry are not routinely performed in the majority of cases. As such, we are unable to provide data or discuss for these analyses in the current study. However, we recognize that the critical role of IHC and molecular markers, such as Ki-67, in enhancing diagnostic accuracy and guiding treatment decisions.

There are reports that Ki-67 evaluation in EUS-FNA samples is a useful tool for excluding G3 tumors, and that immunohistochemical staining for p53 and Ki-67 can improve the sensitivity of EUS-FNA in diagnosing pancreatic adenocarcinoma[9, 10]. Immunostaining in EUS-FNA is therefore extremely important, and a comparative study between FNA and FNB in this context would be highly valuable. A recent meta-analysis also suggested that EUS-FNB might outperform EUS-FNA in terms of obtaining adequate tissue for accurate Ki-67 assessment[11]. This finding indicates that EUS-FNB could provide a more reliable method for tumor grading, thereby enhancing diagnostic precision and potentially reducing the risk of over- or undertreatment. While we were unable to assess these aspects in our current study due to institutional limitations, we agree that further investigations comparing EUS-FNA and EUS-FNB for molecular and IHC adequacy would be highly valuable. We appreciate this suggestion, which highlights an important area for future research.

**Reviewer #2**

**Comment 1 (major):**

Please add the proportion of cases in which the final diagnosis was made based on surgical specimens.

**Responses to the Reviewer’s comments:**

Thank you very much for your valuable comments. The percentage of patients who underwent surgery was 43% in the Lancet needle group and 29.6% in the Franseen needle group, with a statistically significant difference between in the two group (p = 0.02). We added the data to table1, as follow.

We appreciate your insightful suggestion, which has improved the completeness of our study.

| Table 1. Clinical characteristics of the Lancet and Franseen needle groups. | | | |
| --- | --- | --- | --- |
|  | | | |
|  |  |  |  |
|  | **Lancet needle (n=200)** | **Franseen needle (n=115)** | **p value** |
| **Median age (IQR^†^), years** | 69 [60–74] | 71 [66–76] | 0.009 |
| **Sex, male, n (%)** | 115 (57.5) | 62 (53.9) | 0.557 |
| **Median tumor size, mm (IQR^†^)** | 22 [16.1–27.9] | 27 [20.9–32.8] | <0.001 |
| **Location of the lesion, n (%)** |  |  | 0.008 |
| **Head** | 119 (59.5) | 52 (45.2) |  |
| **Body** | 57 (28.5) | 34 (29.6) |  |
| **Tail** | 22 (11.0) | 28 (24.3) |  |
| **All** | 2 (1.0) | 1 (0.9) |  |
| **Puncture route, n (%)** |  |  | 0.001 |
| **Stomach** | 85 (42.5) | 68 (59.1) |  |
| **Duodenal bulb** | 57 (28.5) | 31 (27.0) |  |
| **Duodenal second portion** | 58 (29) | 15 (13.0) |  |
| **Jejumum** | 0 | 1 (1.0) |  |
| **Scopist, expert (%)** | 65 (32.5) | 11 (9.6) | <0.001 |
| **Median number of passes (IQR^†^)** | 2 [2–3] | 2 [1–2] | 0.003 |
| **Adverse events, n (%)** | 3 (1.5) | 3 (2.6) | 0.672 |
| **Surgery** | 86 (43.0) | 34 (29.5) | 0.021 |
| **Final diagnosis, n (%)** |  |  |  |
| **Malignant** | 139 (69.5) | 91 (79.1) | 0.067 |
| **Pancreatic carcinoma** | 126 | 82 |  |
| **Neuroendocrine carcinoma** | 1 | 1 |  |
| **Metastatic cancer** | 5 | 4 |  |
| **Intraductal papillary mucinous with high-grade dysplasia** | 1 | 0 |  |
| **Intraductal papillary mucinous with invasive carcinoma** | 1 | 2 |  |
| **Malignant lymphoma** | 4 | 1 |  |
| **Carcinoma of unknown primary** | 1 | 1 |  |
| **Benign** | 61 (30.5) | 24 (20.9) |  |
| **Autoimmune pancreatitis** | 12 | 11 |  |
| **Neuroendocrine neoplasm** | 15 | 5 |  |
| **Tumor-forming pancreatitis** | 2 | 0 |  |
| **Serous cystic neoplasm** | 5 | 1 |  |
| **Non-specific inflammation** | 14 | 2 |  |
| **Chronic pancreatitis** | 6 | 2 |  |
| **Intraductal papillary mucinous neoplasm** | 2 | 2 |  |
| **Pancreatic pseudocyst** | 2 | 0 |  |
| **Lymphoepitelial cyst** | 0 | 1 |  |
| **Intrapancreatic accessory spleen** | 3 | 0 |  |
| **Adequate sample, %** | 182 (91.0) | 111 (96.5) | 0.069 |
| †: IQR, interquartile range. |  |  |  |

**Comment 2:**

In Table 3, please add the cut-off value for tumor size and number of puncture.

**Responses to the Reviewer’s comments:**

Thank you for your insightful comments. As you pointing out, we calculated the cutoff value. Regarding to number of punctures, an AUC of 0.6 indicates that the model's diagnostic performance was modest, with a 60% probability of correctly distinguishing between cases. This suggests that while two punctures may enhance diagnostic yield to some extent, factors other than the number of punctures, such as needle type, target lesion characteristics, or operator experience, may have a significant influence on diagnostic accuracy. In the other hand, the AUC for tumor size was 0.5 with a cutoff value of 30 mm, indicating that tumor size alone is not a decisive diagnostic factor. We have added this information to Table 3, as follow.

| Table 3. Prognostic factor for histological accuracy. | |  |  |  |  |
| --- | --- | --- | --- | --- | --- |
|  |  |  |  |  |  |
|  | Univariate | |  | Multivariate | |
|  | Crude OR^¶¶^ (95% CI^\|\|\|\|^) | p value |  | Crude OR^¶¶^ (95% CI^\|\|\|\|^) | p value |
| **Age** | 1.00 (0.98–1.03) | 0.656 |  |  |  |
| **Sex, male** | 1.03 (0.59–1.81) | 0.911 |  |  |  |
| **Tumor size** | 1.00 (0.97–1.02) | 0.767 |  |  |  |
| **Puncture route, duodenum** | 1.07 (0.53–0.77) | 0.122 |  | 1.17 (0.83-1.65) | 0.379 |
| **Number of punctures** | 1.47 (1.14–1.88) | 0.003 |  | 1.35 (1.04–1.75) | 0.022 |
| **Needle, Franseen** | 2.29 (1.21–4.35) | 0.011 |  | 1.85 (0.95–3.61) | 0.069 |
| AUC, number of puncture (95% CI) | 61.9 (0.54 - 0.69) | | | | |
| AUC, tumor size (95% CI) | 51.0 (0.44 - 0.58) | | | | |
| ¶¶: OR, odd ratio, \|\|\|\|: CI, confidence interval | | | | | |
|  | | | | | |

**Comment 3:**

The number of EUS-TA experiences of the endoscopists may affect the accuracy of EUS-TA, and the number of EUS-TA experiences shows a significant difference between the two groups in terms of patient background. Therefore, the factor of number of EUS-TA experiences should be added in Table 3.

**Responses to the Reviewer’s comments:**

Thank you for your insightful comments. Previous reports have indicated that there is no difference in diagnostic accuracy based on the skill level of the operator, nor is there a difference in the collection of tissue suitable for comprehensive genomic profiling according to the operator’s level[12]. Furthermore, Mukai et al. reported that there were no technical failures even when practitioners used the Franseen needle[13]. However, the number of endoscopic procedures performed could indeed affect diagnostic accuracy as you pointing out.

In our institution, an expert always supervises the procedure. If a less experienced endoscopist performs the EUS-TA and is unable to obtain a sufficient tissue sample, the expert takes over and performs the puncture. Therefore, we believe this minimizes the impact of operator experience on the results, and for this reason, we did not include it in the logistic regression analysis.
That said, if you believe it is necessary to include this factor in the analysis, we would be happy to consider performing additional analyses.

**Comment 4:**

In Table 3, the puncture route was significantly different in univariate analysis. I recommend adding it as a factor in the multivariate analysis.

**Responses to the Reviewer’s comments:**

Thank you for your valuable comments. As you pointed out, the puncture route may potentially affect the diagnostic accuracy[14]. Previous studies have shown improved histological diagnostic rates with the transgastric route. There was no impact of puncture route differences on the diagnostic accuracy of cytology; however, histological diagnosis showed higher accuracy with the transgastric route compared to the transduodenal route (85.2% vs 76.5) [15]. This is likely due to the greater difficulty of obtaining samples from the transduodenal route, resulting in a smaller sample volume and lower diagnostic accuracy. Taking these reports into account,

we performed a multivariate analysis including the puncture route, and the results are shown in Table 3. We also included both the puncture route and the number of punctures in the multivariate analysis, and it became clear that the Franseen needle remained a significant factor for obtaining a histological diagnosis.

In consideration of your comments, I have added the following to the Discussion section, as follow: **Previous reports have indicated that the number of punctures and puncture route are factors that influence diagnostic accuracy. In the present study, we adjusted for these factors using multivariate analysis, and we found that the diagnostic accuracy of the Franseen needle was still higher compared to the lancet needle** (page 26, line 330-333).

| Table 3. Prognostic factor for histological accuracy. | |  |  |  |  |
| --- | --- | --- | --- | --- | --- |
|  |  |  |  |  |  |
|  | Univariate | |  | Multivariate | |
|  | Crude OR^¶¶^ (95% CI^\|\|\|\|^) | p value |  | Crude OR^¶¶^ (95% CI^\|\|\|\|^) | p value |
| **Age** | 1.00 (0.98–1.03) | 0.656 |  |  |  |
| **Sex, male** | 1.03 (0.59–1.81) | 0.911 |  |  |  |
| **Tumor size** | 1.00 (0.97–1.02) | 0.767 |  |  |  |
| **Puncture route, duodenum** | 1.07 (0.53–0.77) | 0.122 |  | 1.17 (0.83-1.65) | 0.379 |
| **Number of punctures** | 1.47 (1.14–1.88) | 0.003 |  | 1.35 (1.04–1.75) | 0.022 |
| **Needle, Franseen** | 2.29 (1.21–4.35) | 0.011 |  | 1.85 (0.95–3.61) | 0.069 |
| AUC, number of puncture (95% CI) | 61.9 (0.54 - 0.69) | | | | |
| AUC, tumor size (95% CI) | 51.0 (0.44 - 0.58) | | | | |
| ¶¶: OR, odd ratio, \|\|\|\|: CI, confidence interval | | | | | |
|  | | | | | |

**Comment 5:**

In the amount of core tissue and blood contamination section, you mentioned that the cases for evaluation of tissue specimens were randomly selected. Please describe in detail what method was used to randomly select the cases.

**Responses to the Reviewer’s comments:**

Thank you for your insightful comments. This study was conducted as a retrospective design. Due to the inability to collect slides from older cases, we randomly extracted 30 cases from the available slides. We assigned serial numbers to the remaining slides from both the Lancet needle group and the Franseen needle group. Then, we generated a random number table in Excel and selected 30 cases starting from the smallest numbers. This method **“It was not possible to collect slides for all cases, therefore we randomly extracted 30 cases from the available slides. We assigned serial numbers to the remaining slides from both the Lancet needle group and the Franseen needle group. Then, we generated a random number table in Excel and selected 30 cases starting from the smallest numbers.”** has been added to the 'Methods' section on page 10, line 173-176.

**Comment 6 (minor):**

Ref 12 is a protocol article. Please change to PMID: 35151711.

**Responses to the Reviewer’s comments:**

Thank you very much for your valuable comments. We have corrected the citation [3] and referenced the appropriate paper.

**Reviewer #3**

**Comment 1:**

Please change EUS-FNAB in EUS-guided tissue acquisition (EUS-TA).

**Responses to the Reviewer’s comments:**

Thank you for your valuable comments. As you suggested, we have replaced 'EUS-FNAB' with 'EUS-TA'.

**Comment 2:**

In the abstract and in the text, please specify the study setting (academic hospital?)

**Responses to the Reviewer’s comments:**

Thank you for your valuable comments. Since it has already been stated in the methods section that this is a “university hospital”, we have added this information to the abstract, as you pointed out. Please refer to page 2, line 26.

**Comment 3:**

In the abstract, be consistent when you write "diagnostic yield", "sensitivity" or "accuracy"

**Responses to the Reviewer’s comments:**

Thank you for your valuable comments. We have removed the term 'diagnostic yield' and consistently changed it to 'diagnostic accuracy.' Please refer to page 2.

**Comment 4:**

Study aims and endpoints shoud be clearly listed in the Method section. Your "main outcome measure" was "Histological accuracy". What exactly it means? You should refer to standard definitions of outcome measures reported in the AGA white paper (PMID: 29074447). Moreover, the Lancet needle is a cytology needle (FNA), whereas the Franseen needle is a histological needle (FNB). Therefore, comparing the "histological accuracy" may not be the best way. You should compare, as primary outcome" the "diagnostic accuracy" as defined in the AGA white paper.

**Responses to the Reviewer’s comments:**

Thank you for your valuable feedback. As you pointed out, it was necessary to clarify the definition of "histological accuracy." In EUS-TA diagnosis, there are two major categories: "histological diagnosis" and "cytological diagnosis," both of which are typically performed in Japan, with a comprehensive diagnosis based on the results of both methods. In this study, we hypothesized that the FNB needle may have higher histological diagnostic accuracy due to its specific characteristics. Therefore, we chose "histological diagnostic accuracy" as the primary outcome.

While the AGA White Paper[16] does not provide explicit definitions for histological and cytological diagnoses, we have taken your feedback into account and have revised the terminology to "histological diagnostic accuracy" to provide a clearer and more accurate description.

Additionally, we had previously used "accuracy" and "diagnostic yield" interchangeably in the manuscript. In response to your comments, we have standardized the terminology by using "accuracy" throughout the paper to avoid any confusion.

Furthermore, as noted in the AGA White Paper, "Core specimens are obtained typically using FNB needles, may be obtained with FNA needles, and defined by the presence of a tissue sample that allows for histologic and tissue architectural assessment", it is clear that FNA needles can also yield core specimens. Therefore, comparing histological diagnostic accuracy is not problematic. This is consistent with previous studies where FNA needles have been used to compare histological diagnostic accuracy[15]. On the other hand, FNB needles are also commonly used to perform cytology as well as tissue diagnosis[14].

We hope these revisions address your concerns, and we believe the updated terminology and explanations improve the clarity and consistency of the manuscript.

**Comment 5:**

The same as above for the definition of "adequacy".

**Responses to the Reviewer’s comments:**

Thank you for your valuable feedback. In response to your comment regarding the definition of "adequacy," we have revised the definition as follows:
"**An adequate sample was defined as the percentage of lesions sampled in which the obtained material is representative of the target site and possible for diagnosis. Then, the rest were defined as inadequate samples.**" Please refer to page 9, line 158-160.

We believe this revised definition more clearly reflects the criteria for an adequate sample and aligns with standard practices. Thank you again for your insightful suggestion.

**Comment 6:**

In the discussion, you should discuss if you found differences between the two needles for the diagnosis of autoimmune pancreatitis, because there are reports suggesting the Franseec needle being superior to standard needle in this setting (cite PMID: 32397913 and PMID: 31654634).

**Responses to the Reviewer’s comments:**

Thank you for your insightful comments. As you correctly pointed out, the pathological diagnosis of autoimmune pancreatitis (AIP) is indeed challenging, and previous studies have reported that the diagnostic sensitivity of EUS-FNA for AIP cases is relatively low, approximately 43% to 60%. In previous study, the Franceen needle demonstrated superior sensitivity compared to the forward-bevel needle in randomized controlled trials[17] [18]. However, according to the 2018 Japanese Guidelines for Autoimmune Pancreatitis, a histological diagnosis is not mandatory[19]. The diagnosis can be made by considering CT, MRCP, or ERP imaging findings, as well as serum IgG4 levels and the response to steroid therapy. Therefore, our study primarily focused on differentiating between benign and malignant lesions, rather than specifically aiming for a histological diagnosis of AIP.

In our cohort, EUS-TA was performed on 23 patients with suspected AIP. Unfortunately, none of these cases resulted in a final histological diagnosis of AIP. We believe this outcome is likely due to the primary objective of EUS-TA in this study, which was to differentiate between benign and malignant conditions, and we did not actively investigate immunostaining or thorough examination for key histological features of AIP, such as phlebitis, fibrosis, or plasma cell infiltration.

Given these limitations, a direct comparison between the Franceen and Lancet needles in the context of AIP diagnosis was not feasible in our study.

**Comment 7:**

Also, you should discuss about the possibility of performing Ki-67 evaluation on FNA and FNB samples. Doing so, you should mention a recent metanalysis suggested that FNB could outperform FNA (cite PMID: 35863518).

**Responses to the Reviewer’s comments:**

Thank you for your comments. Unfortunately, in our institution, molecular analysis and immunohistochemistry are not routinely performed in the majority of cases, so we are unable to present results in these areas. As such, we are unable to provide data or discuss this aspect in detail. However, we agree that Ki-67 is important as a diagnostic adjunct.

There are reports that Ki-67 evaluation in EUS-FNA samples is a useful tool for excluding G3 tumors, and that immunohistochemical staining for p53 and Ki-67 can improve the sensitivity of EUS-FNA in diagnosing pancreatic adenocarcinoma [9] [10]. Immunostaining in EUS-FNA is therefore extremely important, and a comparative study between FNA and FNB in this context would be highly valuable. A recent meta-analysis also suggested that EUS-FNB might outperform EUS-FNA in terms of obtaining adequate tissue for accurate Ki-67 assessment[11]. This finding indicates that EUS-FNB could provide a more reliable method for tumor grading, thereby enhancing diagnostic precision and potentially reducing the risk of over- or undertreatment.

**Comment 8:**

Finally, a recent metanalysis demonstrated a higher rate of tissue integrity and performance of the wet-suction technique compared with the other aspiration techniques despite a larger amount of blood contamination. You should mention and comment this study in the discussion (PMID: 36657607).

**Responses to the Reviewer’s comments:**

Thank you for your valuable suggestions. I agree that technical factors such as aspiration technique may affect diagnostic results. Recent studies have demonstrated that wet-suction, both with FNB and FNA needles, can provide better tissue integrity, higher cell density, and better diagnostic accuracy compared with standard aspiration and slow pull techniques (PMID: 36657607, 36255027FNA) [5] [6]. As highlighted by Antonio Facciorusso et al., the wet suction technique demonstrates comparable levels of blood contamination to the dry suction technique while achieving high diagnostic accuracy [5]. Specifically, when limited to the Franseen needle, wet suction has been shown to achieve the highest diagnostic accuracy compared to dry suction and non-suction methods. These findings highlight the potential impact of the suction method on EUS-FNAB performance. However, as our study is retrospective in nature, detailed information regarding the aspiration method (e.g., wet suction or suction) was not consistently recorded across all cases. This lack of data precluded us from conducting subgroup analyses based on these technical factors. We acknowledge this as a limitation of our study and have clearly addressed it in the revised manuscript (page 30, line 403-409), “**First, we could not evaluate the relationship between factors such as the utilization of suction method (general suction or wet-suction), however, these factors may potentially influence diagnostic accuracy.**

**Previous reports have indicated that wet suction resulted in blood contamination similar to that of the general suction method, but yielded better results in terms of diagnostic rate and tissue quality. Similarly, the puncture technique such as door knocking method or fanning method may affect diagnostic accuracy; however, due to insufficient data on this factor, it could not be fully evaluated in this study.**”. Furthermore, when the amount of tissue obtained from the first aspiration is small, we try various methods to extract more tissue. Given this background, it is difficult to make a simple comparison of aspiration methods in this retrospective study. We greatly appreciate the reviewer’s insightful comments, which will guide future studies. Moving forward, prospective studies with standardized documentation of technical variables, including suction methods, are warranted to better elucidate their impact on diagnostic outcomes.

**Reviewer #4**

**Comment 1:**

To test continuous variables among two independent groups, unpaired t-tests (parametric tests) or Mann-Whitney tests (non-parametric tests) should be used properly according to the data distribution following normality tests, e.g., Shapiro-Wirk's or Kolmogorov-Smirnov normality tests.

The modern medical statistics professionals recommend that the Fisher's exact test should always be employed to test 2x2 contingency tables even if the sample size is sufficiently large to obtain "exact" test results. If the contingency tables are larger than 2x2, the Fisher-Freeman-Halton test should be used.

They defined minimum p-values as 0.01 (i.e., p-values smaller than 0.01 are written as "p<0.01). The number of significant digits does not comply with the PLOS ONE's regulations clearly written in "Statistical reporting" section to the PLOS ONE submission guideline. Check the posting rules.

Overall, inappropriate statistical processing is noticeable. The authors should consult with biostatistics experts or one of the co-authors should be a biostatistician.

**Responses to the Reviewer’s comments:**

Thank you for your valuable comment. As you pointed out, the statistical analysis in our initial submission was insufficient. In response to your feedback, we conducted a thorough re-analysis with the collaboration of several co-authors, ensuring the statistical methods were robust and comprehensive. Age, size, and the number of punctures were not normally distributed according to the Kolmogorov-Smirnov test, so the Mann-Whitney U test was used for analysis. Additionally, when the contingency table was larger than 2x2, the Fisher-Freeman-Halton test was applied for further analysis. P-values were reported up to three decimal places. We organized the data as much as possible and submitted it again as an Excel file.

The new results did not significantly differ from our initial findings, and therefore, they did not impact the interpretations or discussions presented in the manuscript.

We appreciate your insightful feedback, which has helped us strengthen the rigor of our study. Please let us know if further clarification is required.

**Reviewer #5**

**Comment 1:**

The study is retrospective; not all data could be reliably identified.

**Responses to the Reviewer’s comments:**

Thank you for your valuable comments. As this study is retrospective, not all data could be reliably identified or collected, which may limit the conclusions drawn from some of the analyses. We organized the data as much as possible and submitted it again as an Excel file.

**Comment 2&3:**

The study period is too large; the practice of EUS-FNAB evolved over this period of time, and the use of the two types of needles was not concomitant - the novel type of needle entered in clinical use only in the last 5 years of the study.

The heterogeneity in the study groups is quite large; for example, more than one type of Lancet needle was used, which could affect the overall results. If you have any additional suggestions, please let us know.

**Responses to the Reviewer’s comments:**

Thank you for your insightful comments. We registered all cases of EUS-TA performed at our institution; however, as you pointed out, given the long study period, there have been improvements in the design of lancet needles, which could introduce a significant bias by including older cases. While we considered analyzing EZ Shot and Francine needles separately, the sample sizes were too small for meaningful analysis. To examine this issue, we performed a sub-analysis limited to cases from 2016 onwards (n = 196), when the Franceen needle became available. The diagnostic accuracy of the Lancet needle was 64.3%, and that of the Franseen needle was 85.8%. The diagnostic accuracy of both the Lancet needle and Franseen needle has increased, which may have improved the skills of the technologists and endoscopists. In addition, univariate and multivariate analyses were performed on cases from 2016 onwards, and the tissue diagnostic rate of the FNB needle was shown to be significantly better.

　Regarding to needle type, as you mentioned, the sono tip and expect are lancet needles, while the EZshot3plus is a Menghini needle [7]. However, all of these are considered FNA needles, and it has been reported that the EZ shot3 plus is included in the lancet needle group [8]. The Menghini needle is said to have a sharper lancet tip, which improves puncture performance. Therefore, I believe there is no issue with the classification. Furthermore, if we were to conduct subgroup analysis comparing lancet needles and Menghini needles, the sample size would be very small, making the results less reliable. However, we will consider adding further clarification or subgroup analysis if necessary in the revised manuscript.

**Comment 4:**

The study center has a low volume of procedures. This results from the fact that 315 patients were enrolled over 15 years, which means that a mean of less than 21 patients were enrolled per year in both arms.

**Responses to the Reviewer’s comments:**

Thank you for your valuable feedback. This study includes cases from the early stages of EUS-TA implementation at our institution, during which the number of procedures was quite low. As a result, the average number of cases per year appears smaller. However, we have seen a steady increase in procedures over time, and in recent years, we perform approximately 100 to 150 cases annually. Since this study is limited to initial cases, the numbers are lower than the total yearly procedures. We hope this clarifies the situation. If you have any additional suggestions, please let us know.

**Comment 5:**

The study is single center, in a center where the diagnostic accuracy using the novel types of FNB needles is 10% less than expected as recommended by quality in endoscopy guidelines, therefore the center's endoscopic/histopathologic practice needs to be improved.

**Responses to the Reviewer’s comments:**

Thank you for your comments. The results in Table 2 represent the diagnostic accuracy based solely on histological diagnosis. When cytological findings are also considered, the diagnostic accuracy of the FNB needle increases to 92.9%. This result is not low compared to general diagnostic accuracy. Therefore, we believe that the overall diagnostic accuracy is not low.

| S 1. Diagnostic abilities of a Lancet and Franseen needle. | | | |
| --- | --- | --- | --- |
|  |  |  |  |
|  | **Lancet needle (n = 174)** | **Franseen needle (n = 98)** | **p value** |
| ***Cytology*** |  |  |  |
| Sensitivity, % | 77.2 (0.69-0.84) | 83.5 (0.74-0.91) | 0.284 |
| Specificity, % | 97.9 (0.89-1.00) | 100 (0.93-1.00) | 0.497 |
| PPV^‡^, % | 99.0 (0.95-1.00) | 100 (0.93-1.00) | 1.000 |
| NPV^§^, % | 61.3 (0.50-0.72) | 48.1 (0.29-0.68) | 0.089 |
| Diagnostic accuracy, % | 82.8 (0.76-0.88) | 85.7 (0.77-0.92) | 0.696 |
| ***Combined (Cytology and Histology)*** | |  |  |
| Sensitivity, % | 81.1 (0.73-0.88) | 91.8 (0.84-0.97) | 0.037 |
| Specificity, % | 100 (0.89-1.00) | 100 (0.66-1.00) | 1.000 |
| PPV^‡^, % | 100 (0.95-1.00) | 100 (0.93-1.00) | 1.000 |
| NPV^§^, % | 66.2 (0.80-0.91) | 65.0 (0.41-0.85) | 1.000 |
| Diagnostic accuracy, % | 86.2 (0.80-0.91) | 92.9 (0.86-0.97) | 0.165 |
| §: NPV, Negative predictive value; ‡: PPV, Positive predictive value. | | |  |

**Comment 6:**

On the study overall set of data, a statistical method needed to be used (the inverse probability of treatment weighting method), and it is not clear why. Probably because the study groups are not equivalent.

**Responses to the Reviewer’s comments:**

Thank you for your insightful comments. The propensity score allows for pseudo-randomization of study groups. The usefulness of the propensity score is well-documented in the literature, particularly in retrospective studies where conducting an RCT is challenging. By applying the IPTW method, we aimed to eliminate selection bias and improve the overall quality of the study. Through this adjustment, we were able to achieve pseudo-randomization with respect to factors such as patient age, tumor size, tumor location, puncture site, and the endoscopist's experience, which were previously imbalanced between the groups.

**Comment 7:**

Postive EUS-FAB results alone could classify a patient as positive, which could negatively impact upon the calculation of specificity and accuracy.

**Responses to the Reviewer’s comments:**

Thank you for your comment. As stated in the "True and False Diagnosis" section, the final diagnosis was based on surgical histological results in patients who underwent surgery. In the other hand, for cases without surgery, the true diagnosis was determined based on positive findings from EUS-TA, disease progression, and cancer-related mortality. These were defined based on previous reports[4]. However, we included only those cases where follow-up for at least 12 months was possible, even for those with positive EUS-TA results. I would like to assure you that there were no discrepancies in the results for these cases.

Taking your feedback into account, we have added the detail regarding the 12-month follow-up in the manuscript “**In case where patients did not undergo surgery, positive EUS-TA results, disease progression, along with a follow-up period of 12 months, were considered the final diagnosis**”. Please refer to the Methods section for the revised information page 9, line 163-165.

Additionally, for the cytology evaluation, the diagnosis was made based on the guidelines set forth by the Japanese Society of Cytology. This approach ensured that all decisions were aligned with current standards in cytological practice in Japan. Furthermore, at our hospital, the pathologists who perform cytological diagnoses and histological diagnoses are different doctors, so the diagnosis does not affect the results of either test.

**Comment 8:**

For the evaluation of core tissue and blood contamination it was not possible to collect slides for all cases, therefore only30 cases were randomly selected and the slides were evaluated.

**Responses to the Reviewer’s comments:**

Thank you for your insightful comments. This study was conducted as a retrospective design. Due to the inability to collect slides from older cases, we randomly extracted 30 cases from the available slides. We assigned serial numbers to the remaining slides from both the Lancet needle group and the Franseen needle group. Then, we generated a random number table in Excel and selected 30 cases starting from the smallest numbers. This method **“It was not possible to collect slides for all cases, therefore we randomly extracted 30 cases from the available slides. We assigned serial numbers to the remaining slides from both the Lancet needle group and the Franseen needle group. Then, we generated a random number table in Excel and selected 30 cases starting from the smallest numbers.”** has been added to the 'Methods' section on page 10, line 173-176.

We also acknowledged this point in the Limitations section. Please refer to page 31, line 418-421 for further details.

**Comment 9:**

The study groups are not equivalent: 50 patients (20%) were excluded from the first group and only 1 (1%) from the second group; tumor size was significantly larger in the Franseen needle group than in Lancet needle group (p<0.01); the number of expert endoscopists was significantly higher in first group (p<0.01). The same for location of the tumor, needle puncture route and final diagnoses.

**Responses to the Reviewer’s comments:**

Thank you for your valuable comments. Many of the cases in the Lancet needle group are relatively older, and due to the lack of electronic medical records for those cases, we were unable to gather complete data, leading to an increase in excluded cases. Additionally, there are background biases regarding tumor size, tumor location, puncture route, and endoscopist experience. The size of the tumor may affect the diagnosis due to the ease of puncture or stroke. In this study, there was a significant difference in tumor size, but the median was over 20 mm in both groups, so we believe that there is no significant impact. There is also a significant difference in the puncture route due to the distribution of tumor location in the two groups, but we believe that puncture is more difficult when using an FNB needle because the tail of the pancreas is farther away from the digestive tract. In general, diagnostic accuracy tends to decrease when puncture is difficult, and in the distribution of this study, we believe that the diagnostic accuracy is lower with an FNB needle, considering the tumor location and puncture route.

However, we have addressed these biases as much as possible using the IPTW method, which allows us to simulate randomization and reduce the impact of these background differences.

However, since this is not a fully randomized study, some selection bias may remain. We have acknowledged this limitation in the Limitations section “**Fifth, this was a retrospective study that used the IPTW method. Propensity score analysis is a statistical method for adjusting for selection bias in observational studies and approximates randomized trial approaches.**”. Please refer to page 25, line 417-419 for further details.

**Comment 10:**

The study results are not new. Almost all previous studies, some prospective, concluded that Franseen needle obtained higher amount of tissue, which led to its high diagnostic accuracy.

**Responses to the Reviewer’s comments:**

Thank you for your valuable feedback. As you mentioned, high-quality studies, including RCTs comparing Franseen needles and lancet needles, have already been published. However, in Japan, EUS-TA is evaluated using two diagnostic methods: cytology and histology. There have been no reports evaluating the utility of the Franseen needle using both diagnostic methods, and I believe that we have been able to secure a sufficiently large sample size compared to previous studies. Additionally, comparisons of blood contamination levels have not been conducted. Therefore, I believe that reporting the results of this study will provide valuable insights. I hope you understand this point, and please feel free to share any further opinions or suggestions.

**Comment 11:**

There was no dedicated protocol for cytology using the Fanseen needles, such as touch imprint cytology; the sample is generally processed as a histological sample (i.e. fixed in formalin), and not as a cytological sample.

**Responses to the Reviewer’s comments:**

Thank you for your valuable comments. We had not previously defined the method for cytology. For cytology, the samples prepared by ROSE were later evaluated by a cytotechnologist and a pathologist. Therefore, we have added the sentence, **'The cytological evaluation was based on the samples prepared by ROSE.**' Please refer to page 8, line 135-136. The method for preparing the ROSE samples follows the previously reported protocol[20], and we believe this approach is appropriate.

**Reviewer #6**

**Comment 1:**

(General comment) The manuscript should closely follow RECORD Guideline [https://www.record-statement.org/](https://jpn01.safelinks.protection.outlook.com/?url=https%3A%2F%2Fwww.record-statement.org%2F&data=05%7C02%7C%7C0778f4908cc24054970508dcce5a3ed2%7C84df9e7fe9f640afb435aaaaaaaaaaaa%7C1%7C0%7C638612134285649711%7CUnknown%7CTWFpbGZsb3d8eyJWIjoiMC4wLjAwMDAiLCJQIjoiV2luMzIiLCJBTiI6Ik1haWwiLCJXVCI6Mn0%3D%7C0%7C%7C%7C&sdata=38MsgXn3GxsuSM1lNDgesuVmX%2FV9gYgjsUPhDoHPZlw%3D&reserved=0).

**Responses to the Reviewer’s comments:**

Thank you for your valuable comments. We have carefully reviewed the manuscript to ensure it closely follows the RECORD guidelines. All required items are included, and we have attached the completed checklist for your reference.

**Comment 2:**

(Title) Please avoid the use of abbreviations in the title of the manuscript.

**Responses to the Reviewer’s comments:**

Thank you for your valuable comments. We have revised the title to **“Evaluation of the 22G Franseen needle and 22G Lancet needle for endoscopic ultrasonography-guided tissue acquisition sampling in solid pancreatic lesions: Propensity score weighting**” without using abbreviations. Please review the updated title.

**Comment 3:**

(Abstract) "were evaluated using a scoring system" which scoring system? Please be specific.

**Responses to the Reviewer’s comments:**

Thank you for your valuable comments. We recognize that the previous expression was unclear. We have changed 'evaluated using a scoring system' to 'evaluated using a score.' Due to word count limitations, we were unable to include the specific scoring system in the abstract. However, it has been detailed in the Methods section of the manuscript. **“****It was not possible to collect slides for all cases, therefore we randomly extracted 30 cases from the available slides. We assigned serial numbers to the remaining slides from both the Lancet needle group and the Franseen needle group. Then, we generated a random number table in Excel and selected 30 cases starting from the smallest numbers. The amount of core tissue was scored as: 1 (no material), 2 (a tissue fragment), 3 (a small histological core tissue <×10 objective), and 4 (a large histological core tissue >×10 objective); and blood contamination as: 1 (few), 2 (moderate), and 3 (high) (Fig 1).”** page 10, line 173-180.

**Comment 4:**

(Abstract) "were divided into the Lancet (n=200) and Franseen needle group (n=115)" Which criterion was used?

**Responses to the Reviewer’s comments:**

Thank you for your comment. Due to the word count limitations of the Abstract, we were unable to provide a detailed explanation there. However, we have included a thorough description of the criterion used to divide the groups in the Method section page 7, line113-120. Specifically, we stated: "**Until the Franseen needle (Acquire® FNB needle, Boston Scientific Corporation, Natick, MA, USA) was commercially available, we used a Lancet needle (Expect®, Boston Scientific Corporation, Natick, MA, USA; SonoTip® ProControl, Medi-Globe GmbH, Rohrdorf, Bayern, Germany; EZ Shot3 Plus®, Olympus, Tokyo, Japan). After the Franseen needle was commercially available (starting December 2016), needle selection was based on an endoscopist’s clinical judgement.".**

We hope this addresses your concern. Please let us know if additional clarification is needed.

**Comment 5:**

(General comment) Report the p-values with at least three decimals.

**Responses to the Reviewer’s comments:**

Thank you for your comments. We apologize for overlooking the PLOS ONE guidelines. As per your instructions, we have corrected the P-values accordingly.

**Comment 6:**

(Abstract) "blood contamination scores of 3" it is unclear what a score of 3 means.

**Responses to the Reviewer’s comments:**

Thank you for your comments. The amount of core tissue was scored as: 1 (no material), 2 (a tissue fragment), 3 (a small histological core tissue <×10 objective), and 4 (a large histological core tissue >×10 objective) according to previously reported; and blood contamination as: 1 (few), 2 (moderate), and 3 (high). We could not describe the details about score in the abstract, due to word count limitations. We revised 'blood contamination scores of 3' to 'blood contamination scores of high' for clarity. It has been detailed in the Methods section **“It was not possible to collect slides for all cases, therefore we randomly extracted 30 cases from the available slides. We assigned serial numbers to the remaining slides from both the Lancet needle group and the Franseen needle group. Then, we generated a random number table in Excel and selected 30 cases starting from the smallest numbers. The amount of core tissue was scored as: 1 (no material), 2 (a tissue fragment), 3 (a small histological core tissue <×10 objective), and 4 (a large histological core tissue >×10 objective); and blood contamination as: 1 (few), 2 (moderate), and 3 (high) (Fig 1)”** of the manuscript. page 10, line 173-180.

**Comment 7:**

(Introduction) "was significantly higher than that of Lancet needle" please be specific and write the ranges of the reported values with the associated 95% confidence interval.

**Responses to the Reviewer’s comments:**

Thank you for your valuable comment. As you pointed out, we have added a detailed explanation in the Introduction section, citing the previous report [3] as follows:

"**According to the report by Itonaga et al., the diagnostic accuracy of the Franseen needle was significantly higher than that of the standard needle, with an accuracy of 84.0% (95% CI: 79.0–88.2) compared to 71.2% (95% CI: 65.2–76.6) (p < .001)."**

For further details, please refer to the Introduction section on page 4, line 53-56.

We hope this revision addresses your concern. Please let us know if additional clarification is needed.

**Comment 8:**

(Introduction) "accuracy is limited" please be specific.

**Responses to the Reviewer’s comments:**

Thank you for your comments. The original sentence was unclear, so we have revised it for clarity. We changed the sentence “However, tissue obtained from EUS-FNAB is traditionally evaluated using cytology and histology, which has not been discussed about the details of diagnosis method; cytological, histological or combined accuracy is limited in the Franseen needle and Lancet needle.” to "**However, tissue obtained from EUS-FNAB is traditionally evaluated using cytology and histology, which has not been discussed about the details of diagnosis method such as cytological, histological or combined accuracy in the Franseen needle and Lancet needle.",** please refer to the Introduction section on page 4, line 58-61.

**Comment 9:**

(Introduction) "amounts of tissue core were evaluated in some reports" were comparable? Please be specific.

**Responses to the Reviewer’s comments:**

Thank you for your insightful comment. We have revised the sentence to provide more specific details regarding the comparison of core tissue amounts between needle types. The revised sentence now reads:

**"** **Moreover, it has been reported that the amount of core tissue collected with the Franseen needle is significantly larger than that collected with the Lancet needle, as demonstrated in multiple studies (P < 0.001). However, the amount of blood contamination between the Franseen needle and the Lancet needle has been directly compared in only a few studies**.**"**

For further details, please refer to the introduction on page 4, line 61-67.

We hope this revision addresses your concern and clarifies the specific comparisons mentioned. Please let us know if further adjustments are needed.

Please see these papers as follows; Asokkumar et al. reported that the Franseen needle yielded significantly greater median total tissue area (5.2 mm2 vs. 1.9 mm2, P < 0.001), diagnostic tissue area (2.2 mm2 vs. 0.9 mm2, P = 0.029), and DS area (2 mm2 vs. 0.1 mm2, P = 0.001) than the Lancet needle [2]. Mukai et al. also reported that the amount of tissue obtained with the Franseen needle was significantly greater than that obtained with the End-cut needle (2.13 mm2 vs. 0.45 mm2, P < 0.001) [13]. Bang et al. also reported that median total tissue area (6.1 mm2 (IQR 2.2–9.9) vs. 0.28 mm2 (IQR 0.045–0.93), p<0.0001), tumor (0.68 mm2 (IQR 0.23–2.8) vs. 0.099 mm2 (IQR 0.004–0.30), p<0.0001), fibrosis (3.9 mm2 (IQR 0.5–8.2) vs. 0 (IQR 0–0.11), p<0.0001), residual histology (93.5% vs. 19.6%, p<0.0001), and cell block diagnostic yield (97.8% vs. 82.6% p=0.03) were significantly higher with the Franseen needle compared to the Lancet needle [21].

**Comment 10:**

(Introduction) "would be superior to that of a Lancet needle" explain why.

**Responses to the Reviewer’s comments:**

Thank you for your insightful comments. As mentioned above “Newly shaped needles called “Franseen needles” have been developed for EUS-FNB. Franseen needle can extract a large core tissue sample with few needle passes using a three-pronged cutting edge. Some studies have reported that the accuracy of the Franseen needle was significantly higher than that of Lancet needle because of the higher amount of the core tissue.” in introduction section, we believe that the higher diagnostic accuracy of the Franseen needle may be due to its ability to obtain larger tissue samples, which allows for more reliable histological diagnosis rather than relying solely on cytology from individual cells.

**Comment 11:**

(Introduction) The state of the art is not presented in sufficient details to capture the gaps in the scientific literature.

**Responses to the Reviewer’s comments:**

Thank you for your valuable feedback. As you pointed out, the introduction was unclear, and we have revised it to better address the current gaps and limitations in the literature. We hope the updated version provides a clearer context for our study. Please see page on 4.

**Comment 12:**

(Methods) During 2006 and 2021 changes in needles manufacturing could be seen. Furthermore, different manufacturers had different quality for the same medical device.

**Responses to the Reviewer’s comments:**

Thank you for your valuable comments. As you correctly pointed out, in this study, we utilized three types of needles: EZ Shot 3 Plus, Expect, and ProCore. These needles were chosen due to their widespread use in clinical practice during the study period (2006-2021). However, it is important to note that differences in manufacturing processes and quality between manufacturers may have influenced the performance of these devices.

We added the sentence in the limitation as follow; **the study utilized three types of needles—EZ Shot 3 Plus, Expect, and ProCore—which were selected based on their availability and widespread use during the study period (2006–2021). However, differences in manufacturing processes, quality, and design among these needles may have influenced the results, including diagnostic yield and sample adequacy. Variations in technological advancements over time, such as changes in material composition or needle tip design, could also have introduced performance differences that were not fully controlled in this study.** Please see page 30, line 410-416 in the limitations section.

**Comment 13:**

(Methods) "insufficient data" which data? Please be specific.

**Responses to the Reviewer’s comments:**

Thank you for your comments. We apologize for the lack of detail in the previous version and have now provided a more comprehensive description “**The exclusion criteria were cases with missing data, as mentioned in the data collection section.**”. Specifically, one case was excluded from the Franseen needle group because follow-up was not possible, and 49 cases were excluded from the Lancet needle group because of the lack of information on tumor location, puncture route, number of punctures, endoscopist, or follow-up.

Please refer to page 5, line 83-84 of the Methods section and figure 2.

**Comment 14:**

(Methods) The performance also depend by the experience of the physician. How did you treat this aspect in your study.

**Responses to the Reviewer’s comments:**

Thank you for your insightful comments. Previous reports have indicated that there is no difference in diagnostic accuracy based on the skill level of the operator, nor is there a difference in the collection of tissue suitable for comprehensive genomic profiling according to the operator’s level[12]. Furthermore, Mukai et al. reported that there were no technical failures even when practitioners used the Franseen needle[13]. However, the number of endoscopic procedures performed could indeed affect diagnostic accuracy as you pointing out.

In our institution, an expert always supervises the procedure. If a less experienced endoscopist performs the EUS-TA and is unable to obtain a sufficient tissue sample, the expert takes over and performs the puncture. Therefore, we believe this minimizes the impact of operator experience on the results, and for this reason, we did not include it in the logistic regression analysis.
That said, if you believe it is necessary to include this factor in the analysis, we would be happy to consider performing additional analyses.

**Comment 15:**

(Methods) "All the patients provided written informed consent for the use of their personal data." "This retrospective comparative study was conducted at a single center." It is unclear how in a study with retrospective data collection the patients gave their consent.

**Responses to the Reviewer’s comments:**

Thank you for your valuable comments. In our department, comprehensive research consent is obtained from all patients upon admission. Additionally, for this specific study, we provided information to the patients through an opt-out process, giving them the opportunity to decline participation. This ensures that patients have the chance to refuse the use of their data if they so choose.

**Comment 16:**

(Methods) It is unclear which was the gold standard diagnosis.

**Responses to the Reviewer’s comments:**

Thank you for your valuable comments. As you pointed out, the description of the diagnostic definitions in the Methods section was unclear and ambiguous. However, the Japanese EUS-FNA guidelines do not specify a gold standard for diagnosis. Therefore, we provided detailed explanations in the sections on Malignant and benign lesions definitions and True Diagnosis and False Diagnosis, referring to previous reports. Malignant and benign lesions definitions: We defined pancreatitis, intraductal papillary mucinous neoplasm (IPMN), intraductal papillary mucinous adenoma, serous cystic neoplasm, neuroendocrine tumor (G1, G2), autoimmune pancreatitis, pseudopancreatic cysts, accessory spleen, and lymphoepithelial cysts as benign. We defined high-grade dysplasia and invasive carcinoma It was defined as IPMN, neuroendocrine tumor (G3), malignant lymphoma, metastatic pancreatic cancer, carcinoma of unknown primary origin, and pancreatic cancer as malignant[4].

For True Diagnosis and False Diagnosis, an adequate sample was defined as the percentage of lesions sampled in which the obtained material is representative of the target site and sufficient for diagnosis. Then, the rest were defined as inadequate samples [4, 22]. An adequate sample was defined as the percentage of lesions sampled in which the obtained material is representative of the target site and sufficient for diagnosis. The final diagnosis was based on surgical histological results in patients who underwent surgery. In case where patients did not undergo surgery, positive EUS-TA results, disease progression, along with a follow-up period of 12 months, were considered the final diagnosis[16, 23]. True diagnosis was defined as the coinciding of the final diagnosis and EUS-TA results. Different results were defined as a false diagnosis. Diagnostic accuracy was defined as the percentage of lesions that obtained true diagnosis. Please refer to page 9, line 158-167 for these revisions. These definitions have been referenced from a previous report by Ogura. We believe that our revised definition has few inconsistencies in daily clinical practice and previous reports, and is a general diagnostic definition.

**Comment 17:**

(Methods) Define ROSE abbreviation.

**Responses to the Reviewer’s comments:**

Thank you for your confirmation. ROSE stands for 'rapid onsite evaluation,' and we have specified this abbreviation.

**Comment 18:**

(Methods) "A thin specimen was placed on a slide and air-dried" When? By whom?

**Responses to the Reviewer’s comments:**

Thank you for your question. ROSE is performed by either the endoscopist or the cytotechnologist during the EUS-TA procedure. We have added the sentence, “**by the endoscopist or cytotechnologist during the EUS-TA procedure**” to clarify this. Please refer to page 8, line 133-134 in the Methods section for more details.

**Comment 19:**

(Methods) "30 cases were randomly selected and the slides were checked" this is insufficient. Why 30? The variability in a such a small sample is not representative for the eligible population.

**Responses to the Reviewer’s comments:**

Thank you for your insightful comments. This study was conducted as a retrospective design. Due to the inability to collect slides from older cases, we randomly extracted 30 cases from the available slides. We assigned serial numbers to the remaining slides from both the Lancet needle group and the Franseen needle group. Then, we generated a random number table in Excel and selected 30 cases starting from the smallest numbers. This method has been added to the 'Methods' section on page 10, line 173-176.

We also acknowledged this point in the Limitations section. Please refer to page 31, line 418-421 for further details.

As you point out, the results are not representative of the whole, but the primary endpoint of this study is the histological diagnostic accuracy, not the amount of blood contamination.

30 cases were randomly selected with reference to previous reports, and the amount of blood contamination with FNA and FNB needles was compared. Although this is a small sample, we believe that this is a result that endoscopists would want to know in clinical practice. We present this as a reference when puncturing hypervascular tumors, etc. We hope that readers will understand that this is the result of a small sample and use it as a reference.

**Comment 20:**

(Methods) Define "few", "moderate" and "high".

**Responses to the Reviewer’s comments:**

Thank you for your comment. The definitions for the amount of core tissue and blood contamination have been provided in the "Amount of Core Tissue and Blood Contamination" section as “**It was not possible to collect slides for all cases, therefore we randomly extracted 30 cases from the available slides. We assigned serial numbers to the remaining slides from both the Lancet needle group and the Franseen needle group. Then, we generated a random number table in Excel and selected 30 cases starting from the smallest numbers. The amount of core tissue was scored as: 1 (no material), 2 (a tissue fragment), 3 (a small histological core tissue <×10 objective), and 4 (a large histological core tissue >×10 objective); and blood contamination as: 1 (few), 2 (moderate), and 3 (high) (Fig 1)”** on page 10, line 173-180. As there are currently no standardized or universally accepted measurement methods, we have adopted the definitions proposed in the previous study by Ishigaki et al. However, we acknowledge that this evaluation remains subjective. We appreciate your understanding of this point.

If you have any further suggestions or concerns, we would be happy to address them.

**Comment 21:**

(Methods) "If the sample size was too small, Fisher’s exact test" this is out of statistical knowledge.

**Responses to the Reviewer’s comments:**

Thank you for your insightful comment regarding the statistical analysis. As you correctly pointed out, Fisher's exact test is generally appropriate when the total sample size is 20 or fewer, or when the number of outcomes within each group (both event and non-event) is less than 5. In our analysis, we calculated the expected values under the assumption of no difference between the groups, and none of the expected values were below 5. Therefore, the chi-square test might be appropriate for our statistical analysis.

However, another reviewer also commented as follow; the modern medical statistics professionals recommend that the Fisher's exact test should always be employed to test 2x2 contingency tables even if the sample size is sufficiently large to obtain "exact" test results.

We originally performed the analysis using the Fisher's exact test and have now re-validated our results through recalculation to confirm their correctness. Therefore, we removed the statement that Fisher's exact test was used when sample sizes were small.

We appreciate your valuable input and hope this revision addresses your concern. If you have any further suggestions, we are happy to consider them.

**Comment 22:**

(Methods) Without a gold standard diagnostic your study is just a statistical exercise.

**Responses to the Reviewer’s comments:**

Thank you for your insightful comment. This is related to the point you made in comment 16. We have revised the definition of diagnosis by quoting the definition used in the previous report. We believe that the cited definition of diagnosis is consistent and can be used as a reference in daily practice. Among the eligible cases in this study, many of the final diagnoses were malignant, and AIP and NET accounted for the majority of benign diseases, so we believe that the cited definition of diagnosis is sufficient to provide a basis for diagnosis. Many cases of AIP are treated with steroids, and the response to treatment often leads to an accurate diagnosis. Additionally, NET cases are frequently managed with surgery, which also facilitates precise diagnosis. As the reviewer pointed out, we believe this is an important point that affects the primary endpoint, so we performed statistical analysis again with the revised definition. The results show that FNB needles are useful for diagnosis, and we believe that the reliability has increased. Thank you for your very good point.

**Comment 23:**

(Methods) "Kappa coefficients" is not appropriate according with the described methodology.

**Responses to the Reviewer’s comments:**

Thank you for your comment. The calculation of the κ coefficient was performed using methods based on previous reports. Please refer to the cited literature for further details [24, 25].

**Comment 24:**

(Methods) "receiving the exposure that the patients" define "exposure".

**Responses to the Reviewer’s comments:**

Thank you for your valuable comments. We have clarified that there is potential selection bias by detailing the exposures that patients received, such as needle selection. We revised the manuscript, “**The IPTW was calculated as the inverse of the conditional probability of receiving the specific exposure, defined here as the type of needle (Franceen or Lancet) used during the EUS-TA procedure, that the patients received**” in the method section (Page 11, line 210-213).

**Comment 25:**

(Methods) Generally the SPSS has a one-year license and 21 is far from the current version.

**Responses to the Reviewer’s comments:**

Thank you for your comment. I checked again the version of SPSS that can be used in our laboratory and it was version 26.0, the 2019 model. I also downloaded the latest version of R, V2.9-1, and recalculated everything. Unfortunately, due to lack of funds, our laboratory is unable to update to the latest version of SPSS every year. Therefore, we use an older version. I would appreciate your understanding on this point.

**Comment 26:**

(Methods) It is unclear how age and sex "might influence the diagnostic".

**Responses to the Reviewer’s comments:**

Generally, pancreatic tumors are influenced by age and gender, and we have also examined these factors through univariate analysis. The results of the analysis are included in the results section “**The median age was 69 years in the Franseen needle group and 71 years in the Lancet needle group, with the majority of participants being men (57.5% vs. 53.9%), showing no significant difference**” in page 13, line 230-232. Furthermore, we added this sentence in the prognostic factor analyses for histological accuracy section “**Age, gender, tumor size, and puncture route did not influence histological diagnostic accuracy.**”. Please see on the result section page 16, line 269-270.

**Comment 26:**
(Methods) The details presented in this section does not allow the reproduction/replication.

**Responses to the Reviewer’s comments:**

There were various comments, and as you mentioned, it is possible that the results were not reproducible. Therefore, we conducted a statistical review again and confirmed that there are no issues with the results.

**Comment 27:**
(Methods) It is unclear who chose the type of needle.

**Responses to the Reviewer’s comments:**

Thank you for your valuable comments. The selection of the needle is left to the discretion of the attending endoscopists in charge of cases, and this is described **“needle selection was based on an endoscopist’s clinical judgement**” in the 'Endoscopic Procedure' section of the Methods on page 7, line 119-120.

**Comment 28:**

(Methods) Please provide the date for ethical approval.

**Responses to the Reviewer’s comments:**

Thank you for your valuable comments. The approval date is March 9, 2022, and we have added this information to the Methods section (Page 5, line 90).

**Comment 29:**

(Results) "followed up" no information regarding the follow up was provided in the Methods section.

**Responses to the Reviewer’s comments:**

Thank you for your valuable comments. Although we usually conduct follow-up after EUS-TA, cases where follow-up was not possible, such as patients transferring to another hospital within six months, were excluded. Therefore, the sentence was revised as ' **In case where patients did not undergo surgery, positive EUS-TA results, disease progression, along with a follow-up period of 12 months, were considered the final diagnosis** '. Please see on page 9, line 162-165.

**Comment 30:**

(Results) IQR with round brackets means that the values are not included in the range. Is this correct?

**Responses to the Reviewer’s comments:**

Thank you for your valuable comments. You are correct. I have corrected it by changing the round brackets to square brackets.

**Comment 31:**

(Results) The point estimators in table 2 must have the 95% CI

**Responses to the Reviewer’s comments:**

Thank you for your valuable comments. We revised manuscript of Results section as below.

| Table 2. Histological diagnostic abilities of a Lancet and Franseen needle. | | | |
| --- | --- | --- | --- |
|  |  |  |  |
|  | **Lancet needle (n = 174)** | **Franseen needle (n = 98)** | **p value** |
| Sensitivity, % (95% CI) | 59.8 (0.51-0.68) | 82.4 (0.73-0.90) | <0.001 |
| Specificity, % (95% CI) | 100 (0.89-1.00) | 100 (0.66-1.00) | 1 |
| PPV^‡^, % (95% CI) | 100 (0.93-1.00) | 100 (0.92-1.00) | 1 |
| NPV^§^, % (95% CI) | 48.0 (0.38-0.58) | 46.4 (0.28-0.66) | 0.887 |
| Diagnostic accuracy, % (95% CI) | 70.7 (0.63-0.77) | 84.7 (0.76-0.91) | 0.026 |
| §: NPV, Negative predictive value; ‡: PPV, Positive predictive value. | | |  |

**Comment 32:**

(Results) How the variables were selected for multivariate analysis. Why route is not in the model.

**Responses to the Reviewer’s comments:**

Thank you for your insightful comments. When there are too many explanatory variables, the reliability (reproducibility) of the analysis decreases, and the interpretation of results becomes more difficult. Therefore, it is generally preferable to use fewer explanatory variables. For this reason, it is recommended to narrow down explanatory variables by referring to previous studies. However, as you pointed out, the puncture route may potentially affect the diagnostic accuracy. Previous studies have shown improved histological diagnostic rates with the transgastric route. There was no impact of puncture route differences on the diagnostic accuracy of cytology (76.5%); however, histological diagnosis showed higher accuracy (86.2%) with the transgastric route compared to the transduodenal route[15]. This is likely due to the greater difficulty of obtaining samples from the transduodenal route, resulting in a smaller sample volume and lower diagnostic accuracy. Taking these reports into account, we performed a multivariate analysis including the puncture route, and the results are shown in Table 3. We also included both the puncture route and the number of punctures in the multivariate analysis, and it became clear that the Franseen needle remained a significant factor for obtaining a histological diagnosis.

| Table 3. Prognostic factor for histological accuracy. | |  |  |  |  |
| --- | --- | --- | --- | --- | --- |
|  |  |  |  |  |  |
|  | Univariate | |  | Multivariate | |
|  | Crude OR^¶¶^ (95% CI^\|\|\|\|^) | p value |  | Crude OR^¶¶^ (95% CI^\|\|\|\|^) | p value |
| **Age** | 1.00 (0.98–1.03) | 0.656 |  |  |  |
| **Sex, male** | 1.03 (0.59–1.81) | 0.911 |  |  |  |
| **Tumor size** | 1.00 (0.97–1.02) | 0.767 |  |  |  |
| **Puncture route, duodenum** | 1.07 (0.53–0.77) | 0.122 |  | 1.17 (0.83-1.65) | 0.379 |
| **Number of punctures** | 1.47 (1.14–1.88) | 0.003 |  | 1.35 (1.04–1.75) | 0.022 |
| **Needle, Franseen** | 2.29 (1.21–4.35) | 0.011 |  | 1.85 (0.95–3.61) | 0.069 |
| AUC, number of puncture (95% CI) | 61.9 (0.54 - 0.69) | | | | |
| AUC, tumor size (95% CI) | 51.0 (0.44 - 0.58) | | | | |
| ¶¶: OR, odd ratio, \|\|\|\|: CI, confidence interval | | | | | |
|  | | | | | |

**Comment 33:**

(Results) Also report the 95% CI for κ-value.

**Responses to the Reviewer’s comments:**

Thank you for your valuable comments. We revised the results section of the manuscript as We confirmed poor inter-and agreement (κ-value=0.22, 95% CI: 0.02-0.41) and moderate intra-observer agreement (κ-value=0.54, 95% CI: 0.39-0.69) for the score. (Page 24, line 289-291).

**Comment 34:**

(Results) List adverse effect for each needle with its distribution.

**Responses to the Reviewer’s comments:**

Thank you for your valuable comments. We listed the adverse event for each needle on the table 1.

| Table 1. Clinical characteristics of the Lancet and Franseen needle groups. | | | |
| --- | --- | --- | --- |
|  | | | |
|  |  |  |  |
|  | **Lancet needle (n=200)** | **Franseen needle (n=115)** | **p value** |
| **Median age (IQR^†^), years** | 69 [60–74] | 71 [66–76] | 0.009 |
| **Sex, male, n (%)** | 115 (57.5) | 62 (53.9) | 0.557 |
| **Median tumor size, mm (IQR^†^)** | 22 [16.1–27.9] | 27 [20.9–32.8] | <0.001 |
| **Location of the lesion, n (%)** |  |  | 0.008 |
| **Head** | 119 (59.5) | 52 (45.2) |  |
| **Body** | 57 (28.5) | 34 (29.6) |  |
| **Tail** | 22 (11.0) | 28 (24.3) |  |
| **All** | 2 (1.0) | 1 (0.9) |  |
| **Puncture route, n (%)** |  |  | 0.001 |
| **Stomach** | 85 (42.5) | 68 (59.1) |  |
| **Duodenal bulb** | 57 (28.5) | 31 (27.0) |  |
| **Duodenal second portion** | 58 (29) | 15 (13.0) |  |
| **Jejumum** | 0 | 1 (1.0) |  |
| **Scopist, expert (%)** | 65 (32.5) | 11 (9.6) | <0.001 |
| **Median number of passes (IQR^†^)** | 2 [2–3] | 2 [1–2] | 0.003 |
| **Adverse events, n (%)** | 3 (1.5) | 3 (2.6) | 0.672 |
| **Infection** | 3 (1.5) | 0 |  |
| **Pancreatitis** | 0 | 3 (2.6) |  |
| **Surgery** | 86 (43.0) | 34 (29.5) | 0.021 |
| **Final diagnosis, n (%)** |  |  |  |
| **Malignant** | 139 (69.5) | 91 (79.1) | 0.067 |
| **Pancreatic carcinoma** | 126 | 82 |  |
| **Neuroendocrine carcinoma** | 1 | 1 |  |
| **Metastatic cancer** | 5 | 4 |  |
| **Intraductal papillary mucinous with high-grade dysplasia** | 1 | 0 |  |
| **Intraductal papillary mucinous with invasive carcinoma** | 1 | 2 |  |
| **Malignant lymphoma** | 4 | 1 |  |
| **Carcinoma of unknown primary** | 1 | 1 |  |
| **Benign** | 61 (30.5) | 24 (20.9) |  |
| **Autoimmune pancreatitis** | 12 | 11 |  |
| **Neuroendocrine neoplasm** | 15 | 5 |  |
| **Tumor-forming pancreatitis** | 2 | 0 |  |
| **Serous cystic neoplasm** | 5 | 1 |  |
| **Non-specific inflammation** | 14 | 2 |  |
| **Chronic pancreatitis** | 6 | 2 |  |
| **Intraductal papillary mucinous neoplasm** | 2 | 2 |  |
| **Pancreatic pseudocyst** | 2 | 0 |  |
| **Lymphoepitelial cyst** | 0 | 1 |  |
| **Intrapancreatic accessory spleen** | 3 | 0 |  |
| **Adequate sample, %** | 182 (91.0) | 111 (96.5) | 0.069 |
| †: IQR, interquartile range. |  |  |  |

**Comment 35:**

(Results) The quality of the figure are low.

**Responses to the Reviewer’s comments:**

Thank you for your valuable comments. I have enhanced the quality of the figure and uploaded it again. Please review it once more.

**Comment 36:**

(Discussion) I was not able to see any reference to your own results (tables).

**Responses to the Reviewer’s comments:**

Thank you for your comment. While we had discussed our results in the discussion section, as you pointed out, it was unclear which specific results were being referenced. To address this, we have added table numbers following the relevant discussions for clarity. Please review the revised discussion section.

**Comment 37:**

(Discussion) Put the limitations in the order of their importance.

**Responses to the Reviewer’s comments:**

Thank you for your valuable comments. We revised the limitations in the order of their importance.

**Reviewer #7**

**Comment 1:**

-In the section “true and false diagnosis” the inadequate sample definition is confounding (line 143). Not adequate and not accurate are different definitions, as correctly signed in the following article paragraphs.

**Responses to the Reviewer’s comments:**

Thank you for your valuable comments. I mistakenly confused the definition of an inadequate sample with that of a misdiagnosis. I have corrected this and revised the definition as follows in the method section: "**An adequate sample was defined as one that could be evaluated histologically, and an inadequate sample was defined as one with an insufficient quantity for histological diagnosis**." (Page 9, line 158-160). We performed statistical analysis again based on this definition, but there was no significant difference in the results. Please see Table 1-3.

**Comment 2:**

-in the discussion the impact of the size of the lesion and the benign nature (especially Autoimmune pancreatitis) should be cited.

**Responses to the Reviewer’s comments:**

Thank you for your insightful comments. As you correctly pointed out, the pathological diagnosis of autoimmune pancreatitis (AIP) is indeed challenging, and previous studies have reported that the diagnostic sensitivity of EUS-FNA for AIP cases is relatively low, approximately 43% to 60%. In previous study, the Franceen needle demonstrated superior sensitivity compared to the forward-bevel needle in randomized controlled trials[17] [18]. However, according to the 2018 Japanese Guidelines for Autoimmune Pancreatitis, a histological diagnosis is not mandatory[19]. The diagnosis can be made by considering CT, MRCP, or ERP imaging findings, as well as serum IgG4 levels and the response to steroid therapy. Therefore, our study primarily focused on differentiating between benign and malignant lesions, rather than specifically aiming for a histological diagnosis of AIP.

In our cohort, EUS-TA was performed on 23 patients with suspected AIP. Unfortunately, none of these cases resulted in a final histological diagnosis of AIP. We believe this outcome is likely due to the primary objective of EUS-TA in this study, which was to differentiate between benign and malignant conditions, and we did not actively investigate immunostaining or thorough examination for key histological features of AIP, such as phlebitis, fibrosis, or plasma cell infiltration.

Given these limitations, a direct comparison between the Franceen and Lancet needles in the context of AIP diagnosis was not feasible in our study.

Regarding tumor size, we observed that tumors in the Franseen needle group were larger than those in the Lancet needle group. Generally, FNB needles tend to have lower puncture quality for smaller tumors, leading to a preference for Lancet needles in such cases. This resulted in selection bias, which we mitigated by employing the IPTW method.

Previous studies have reported conflicting findings on the relationship between tumor size and diagnostic accuracy. While some studies suggest that smaller tumors are associated with lower diagnostic accuracy regardless of whether FNA or FNB needles are used [14] [26], others report no significant impact. The AGA White Paper indicates that for FNA needles, high diagnostic accuracy can be achieved for tumors larger than 20 mm with four punctures. However, in our study, we did not classify tumors using a 20 mm threshold, and the median number of punctures was two. These factors may explain why similar results were not observed in our analysis.

In our study, the area under the curve (AUC) for tumor size was 0.5, with a cutoff value of 30 mm. These findings indicate that tumor size did not influence diagnostic accuracy in our analysis. The potential impact of puncture techniques, such as fanning or door-knocking methods, which were not evaluated in this retrospective study, could also contribute to these results.

We have addressed this point in the Discussion section as follows:
" **Regarding tumor size and diagnostic accuracy, some studies have shown that diagnostic accuracy is higher in larger tumors, however, others have found no significant difference. According to the AGA White Paper, tumor sizes exceeding 20 mm with four punctures can achieve high diagnostic accuracy. However, in our study, tumor size did not affect diagnostic accuracy. This may be attributed to the number of punctures or puncture techniques (e.g., fanning or door-knocking methods), which were not evaluated in this retrospective study**."

For further details, please refer to the Discussion section on page 26, line 333-339.

We hope this revision addresses your concerns. Please let us know if additional clarification is required.

**Comment 3:**

-please correct the typo Franssen (line 308).
The article may be accepted after minor revisions.

**Responses to the Reviewer’s comments:**

Thank you for your valuable comments. I revised the manuscript.

1.	Kovacevic B, Toxvaerd A, Klausen P, Larsen MH, Grutzmeier S, Detlefsen S, et al. Tissue amount and diagnostic yield of a novel franseen EUS-FNB and a standard EUS-FNA needle-A randomized controlled study in solid pancreatic lesions. Endosc Ultrasound. 2023;12(3):319-25.

2.	Asokkumar R, Yung Ka C, Loh T, Kah Ling L, Gek San T, Ying H, et al. Comparison of tissue and molecular yield between fine-needle biopsy (FNB) and fine-needle aspiration (FNA): a randomized study. Endosc Int Open. 2019;7(8):E955-E63.

3.	Itonaga M, Yasukawa S, Fukutake N, Ogura T, Asada M, Shimokawa T, et al. Comparison of 22-gauge standard and Franseen needles in EUS-guided tissue acquisition for diagnosing solid pancreatic lesions: a multicenter randomized controlled trial. Gastrointestinal Endoscopy. 2022;96(1):57-66.e2.

4.	Matsuno J, Ogura T, Kurisu Y, Miyano A, Imanishi M, Onda S, et al. Prospective comparison study of franseen needle and standard needle use for pancreatic lesions under EUS guidance. Endosc Ultrasound. 2019;8(6):412-7.

5.	Facciorusso A, Crino SF, Ramai D, Madhu D, Fugazza A, Carrara S, et al. Comparative diagnostic performance of different techniques for EUS-guided fine-needle biopsy sampling of solid pancreatic masses: a network meta-analysis. Gastrointest Endosc. 2023;97(5):839-48 e5.

6.	Chen TY, Cao JW, Jin C, Ji Y, Zhong L, Wang LM, et al. Comparison of specimen quality among the standard suction, slow-pull, and wet suction techniques for EUS-FNA: A multicenter, prospective, randomized controlled trial. Endosc Ultrasound. 2022;11(5):393-400.

7.	Young Bang J, Krall K, Jhala N, Singh C, Tejani M, Arnoletti JP, et al. Comparing Needles and Methods of Endoscopic Ultrasound-Guided Fine-Needle Biopsy to Optimize Specimen Quality and Diagnostic Accuracy for Patients With Pancreatic Masses in a Randomized Trial. Clin Gastroenterol Hepatol. 2021;19(4):825-35 e7.

8.	Matsunami Y, Itoi T, Tsuchiya T, Ishii K, Tanaka R, Tonozuka R, et al. Objective evaluation of the resistance forces of 22-gauge EUS-FNA and fine-needle biopsy needles. Endosc Ultrasound. 2023;12(2):251-8.

9.	Diaz Del Arco C, Diaz Perez JA, Ortega Medina L, Sastre Valera J, Fernandez Acenero MJ. Reliability of Ki-67 Determination in FNA Samples for Grading Pancreatic Neuroendocrine Tumors. Endocr Pathol. 2016;27(4):276-83.

10.	Jahng AW, Reicher S, Chung D, Varela D, Chhablani R, Dev A, et al. Staining for p53 and Ki-67 increases the sensitivity of EUS-FNA to detect pancreatic malignancy. World J Gastrointest Endosc. 2010;2(11):362-8.

11.	Tacelli M, Bina N, Crino SF, Facciorusso A, Celsa C, Vanni AS, et al. Reliability of grading preoperative pancreatic neuroendocrine tumors on EUS specimens: a systematic review with meta-analysis of aggregate and individual data. Gastrointest Endosc. 2022;96(6):898-908 e23.

12.	Ishigaki K, Nakai Y, Endo G, Kurihara K, Ishida K, Tange S, et al. Feasibility of comprehensive genomic profiling using endoscopic ultrasound-guided tissue acquisition with a 22-gauge Franseen needle. DEN Open. 2024;4(1):e365.

13.	Mukai S, Itoi T, Yamaguchi H, Sofuni A, Tsuchiya T, Tanaka R, et al. A retrospective histological comparison of EUS-guided fine-needle biopsy using a novel franseen needle and a conventional end-cut type needle. Endosc Ultrasound. 2019;8(1):50-7.

14.	Ishigaki K, Nakai Y, Sasahira N, Sugimori K, Kitamura K, Iwai T, et al. A prospective multicenter study of endoscopic ultrasound-guided fine needle biopsy using a 22-gauge Franseen needle for pancreatic solid lesions. J Gastroenterol Hepatol. 2021;36(10):2754-61.

15.	Nagai T, Ishida K, Machida H, Takada-Owada A, Onozaki M, Noda S, et al. Usefulness of cytological diagnosis in pancreatic endoscopic ultrasound-guided fine needle aspiration or biopsy: Comparison with histological diagnosis and relationship with puncture route and sample acquisition method. Diagn Cytopathol. 2023;51(8):467-74.

16.	Wani S, Muthusamy VR, McGrath CM, Sepulveda AR, Das A, Messersmith W, et al. AGA White Paper: Optimizing Endoscopic Ultrasound-Guided Tissue Acquisition and Future Directions. Clin Gastroenterol Hepatol. 2018;16(3):318-27.

17.	Kurita A, Yasukawa S, Zen Y, Yoshimura K, Ogura T, Ozawa E, et al. Comparison of a 22-gauge Franseen-tip needle with a 20-gauge forward-bevel needle for the diagnosis of type 1 autoimmune pancreatitis: a prospective, randomized, controlled, multicenter study (COMPAS study). Gastrointest Endosc. 2020;91(2):373-81 e2.

18.	Abdelghani YA, Arisaka Y, Masuda D, Takii M, Ashida R, Makhlouf MM, et al. Bile aspiration cytology in diagnosis of bile duct carcinoma: factors associated with positive yields. J Hepatobiliary Pancreat Sci. 2012;19(4):370-8.

19.	Okazaki K, Kawa S, Kamisawa T, Ikeura T, Itoi T, Ito T, et al. Amendment of the Japanese consensus guidelines for autoimmune pancreatitis, 2020. J Gastroenterol. 2022;57(4):225-45.

20.	Hayashi T, Ishiwatari H, Yoshida M, Ono M, Sato T, Miyanishi K, et al. Rapid on-site evaluation by endosonographer during endoscopic ultrasound-guided fine needle aspiration for pancreatic solid masses. J Gastroenterol Hepatol. 2013;28(4):656-63.

21.	Bang JY, Hebert-Magee S, Navaneethan U, Hasan MK, Hawes R, Varadarajulu S. EUS-guided fine needle biopsy of pancreatic masses can yield true histology. Gut. 2018;67(12):2081-4.

22.	Varadarajulu S, Tamhane A, Eloubeidi MA. Yield of EUS-guided FNA of pancreatic masses in the presence or the absence of chronic pancreatitis. Gastrointest Endosc. 2005;62(5):753, 28-36; quiz 1.

23.	Ramai D, Singh J, Kani T, Barakat M, Chandan S, Brooks O, et al. Wet- versus dry-suction techniques for EUS-FNA of solid lesions: A systematic review and meta-analysis. Endoscopic Ultrasound: Spring Media; 2021. p. 319-24.

24.	Koo TK, Li MY. A Guideline of Selecting and Reporting Intraclass Correlation Coefficients for Reliability Research. J Chiropr Med. 2016;15(2):155-63.

25.	Nagami Y, Tominaga K, Machida H, Nakatani M, Kameda N, Sugimori S, et al. Usefulness of non-magnifying narrow-band imaging in screening of early esophageal squamous cell carcinoma: a prospective comparative study using propensity score matching. Am J Gastroenterol. 2014;109(6):845-54.

26.	Siddiqui AA, Brown LJ, Hong SK, Draganova-Tacheva RA, Korenblit J, Loren DE, et al. Relationship of pancreatic mass size and diagnostic yield of endoscopic ultrasound-guided fine needle aspiration. Dig Dis Sci. 2011;56(11):3370-5.
