# Supplementary material for: Evaluation of the 22G Franseen needle and 22G Lancet needle for endoscopic ultrasonography-guided tissue acquisition sampling in solid pancreatic lesions: Propensity score weighting
Source: PLoS One. 2025 May 16;20(5):e0322857. doi: 10.1371/journal.pone.0322857 (PMC12084063; doi:10.1371/journal.pone.0322857)
Supplement: Attachment — Submitted filename: response to reviewers R2.docx [file pone.0322857.s007.docx]

**Feb 4, 2025**

**Emily Chenette**

**Editors-in-Chief**

**Associate Editors**

***PLOS ONE***

Dear Editor

We appreciate the opportunity to submit a revised version of our manuscript entitled, “**Evaluation of the 22G Franseen needle and 22G Lancet needle for endoscopic ultrasonography-guided tissue acquisition sampling in solid pancreatic lesions: Propensity score weighting.**” (manuscript ID: PONE-D-24-32776R2) for consideration for publication in *PLOS ONE*.

We believe that we have addressed all concerns raised by the reviewers as detailed in the accompanying point-by-point responses.

All authors concur with the submission of this manuscript. We ascertain that none of the data in this manuscript have been previously reported, nor is the manuscript under consideration for publication elsewhere.

We hope that *PLOS ONE* now finds our manuscript suitable for publication. We appreciate your consideration of our work.

Sincerely Yours,

Hirotsugu Maruyama

Hirotsugu Maruyama, M.D. PhD.

Department of Gastroenterology

Osaka City University Graduate School of Medicine

1-4-3, Asahimachi, Abeno-ku, Osaka-City, Osaka, 545-8585, Japan

e-mail to; hiromaruyama99@gmail.com

Phone: +81-6-6645-3811

FAX: +81-6-6645-3813

**Point-by-Point Responses (PONE-D-24-32776R2)**

**Dear Editor and Reviewers,**

Thank you for your valuable feedback and for reviewing our manuscript. We sincerely appreciate your constructive comments, which have helped us improve the quality of our paper. We have carefully addressed all the comments and revised the manuscript accordingly.

Below, we provide our detailed responses to each comment and highlight the corresponding changes in the manuscript.

**Reviewer #4's comment:**

"One minor correction: in P11L193, 'should be used.' should be 'was used.'"

**Response:**

Thank you for your suggestion. We have corrected this phrase accordingly (P11L193).

Regarding the reference list, we have carefully reviewed all cited papers and confirmed that no retracted papers are included.

We have carefully reviewed the statistical processing section, and as Reviewer #4 has acknowledged, the necessary corrections have already been made appropriately in the previous revision. Given that both Reviewer #1 and Reviewer #4 have confirmed that our statistical analysis is now appropriate and rigorous, we believe that no further modifications are required in this regard.

If the Editor still requires additional revisions related to statistical processing, we would appreciate specific guidance on the necessary changes.

We hope that the revisions adequately address your concerns.

Thank you again for your time and effort in reviewing our manuscript.
