# Supplementary material for: Evaluation of the 22G Franseen needle and 22G Lancet needle for endoscopic ultrasonography-guided tissue acquisition sampling in solid pancreatic lesions: Propensity score weighting
Source: PLoS One. 2025 May 16;20(5):e0322857. doi: 10.1371/journal.pone.0322857 (PMC12084063; doi:10.1371/journal.pone.0322857)
Supplement: Attachment — Submitted filename: response to reviewers R3.docx [file pone.0322857.s008.docx]

**Mar 24, 2025**

**Emily Chenette**

**Editors-in-Chief**

**Associate Editors**

***PLOS ONE***

Dear Editor

We appreciate the opportunity to submit a revised version of our manuscript entitled, “**Evaluation of the 22G Franseen needle and 22G Lancet needle for endoscopic ultrasonography-guided tissue acquisition sampling in solid pancreatic lesions: Propensity score weighting.**” (manuscript ID: PONE-D-24-32776R3) for consideration for publication in *PLOS ONE*.

We believe that we have addressed all concerns raised by the reviewers as detailed in the accompanying point-by-point responses.

All authors concur with the submission of this manuscript. We ascertain that none of the data in this manuscript have been previously reported, nor is the manuscript under consideration for publication elsewhere.

We hope that *PLOS ONE* now finds our manuscript suitable for publication. We appreciate your consideration of our work.

Sincerely Yours,

Hirotsugu Maruyama

Hirotsugu Maruyama, M.D. PhD.

Department of Gastroenterology

Osaka City University Graduate School of Medicine

1-4-3, Asahimachi, Abeno-ku, Osaka-City, Osaka, 545-8585, Japan

e-mail to; hiromaruyama99@gmail.com

Phone: +81-6-6645-3811

FAX: +81-6-6645-3813

**Point-by-Point Responses (PONE-D-24-32776R3)**

**Reviewer #2's comment:**

**2. In Table 3, please add the cut-off value for tumor size and number of punctures.**

**Response:**

I apologize for not responding adequately to your previous comments. I had not provided sufficient details regarding the cut-off value, therefore I would like to address them again as follows.

As you pointing out, we calculated the cut-off value. Regarding number of punctures, the cut-off value was three and an AUC of 0.6 indicates that the model's diagnostic performance was modest, with a 60% probability of correctly distinguishing between cases. This suggests that while three punctures may enhance diagnostic yield to some extent, factors other than the number of punctures, such as needle type, target lesion characteristics, or operator experience, may have a significant influence on diagnostic accuracy. In the other hand, the AUC for tumor size was 0.5 with a cut-off value of 29 mm, indicating that tumor size alone is not a decisive diagnostic factor. Additionally, I have included information about the cut-off value in Table 3. Please review the revisions.

| Table 3. Prognostic factor for histological diagnostic accuracy. | |  |  |  |  |
| --- | --- | --- | --- | --- | --- |
|  |  |  |  |  |  |
|  | Univariate | |  | Multivariate | |
|  | Crude OR^¶¶^ (95% CI^\|\|\|\|^) | p value |  | Crude OR^¶¶^ (95% CI^\|\|\|\|^) | p value |
| **Age** | 1.00 (0.98–1.03) | 0.656 |  |  |  |
| **Sex, male** | 1.03 (0.59–1.81) | 0.911 |  |  |  |
| **Tumor size** | 1.00 (0.97–1.02) | 0.767 |  |  |  |
| **Expert** | 1.21 (0.64–2.29) | 0.566 |  |  |  |
| **Puncture route, duodenum** | 1.07 (0.53–0.77) | 0.122 |  | 1.17 (0.83-1.65) | 0.379 |
| **Number of punctures** | 1.47 (1.14–1.88) | 0.003 |  | 1.35 (1.04–1.75) | 0.022 |
| **Needle, Franseen** | 2.29 (1.21–4.35) | 0.011 |  | 1.85 (0.95–3.61) | 0.069 |
| **Cut-off value, AUC (95% CI)** |  | | | | |
| Number of punctures | 3, 61.9 (0.54 - 0.69) | | | | |
| Tumor size, (mm) | 29, 51.0 (0.44 - 0.58) | | | | |
| ¶¶: OR, odd ratio, \|\|\|\|: CI, confidence interval | | | | | |
|  | | | | | |

**3. The number of EUS-TA experiences of the endoscopists may affect the accuracy of EUS-TA, and the number of EUS-TA experiences shows a significant difference between the two groups in terms of patient background. Therefore, the factor of number of EUS-TA experiences should be added in Table 3.**

**Response:**

I apologize for not responding adequately to your previous comments, therefore I would like to address them again as follows.

Thank you for your insightful comments. Previous reports have indicated that there is no difference in diagnostic accuracy based on the skill level of the operator, nor is there a difference in the collection of tissue suitable for comprehensive genomic profiling according to the operator’s level. Furthermore, Mukai et al. reported that there were no technical failures even when practitioners used the Franseen needle. However, the number of endoscopic procedures performed could indeed affect diagnostic accuracy as you pointing out.

We performed both univariable (OR, 1.204; 95% CI, 0.64-2.29; p=0.566) and multivariable analyses (OR, 0.89; 95% CI, 0.45-1.75; p=0.733) to assess whether expertise influenced accuracy, however, no significant results indicating an impact on diagnosis were obtained. In our institution, an expert always supervises the procedure. If a less experienced endoscopist performs the EUS-TA and is unable to obtain a sufficient tissue sample, the expert takes over and performs the puncture. Therefore, we believe this minimizes the impact of operator experience on the results. Additionally, we were concerned that increasing the number of variables could reduce the precision of the multivariable analysis. Therefore, in Table 3 of the manuscript, we have included only the results of the univariable analysis. Then, we added this sentence into the discussion section ‘Subsequently, we evaluated the impact of expert experience on diagnostic accuracy. Previous reports suggest that the skill level of the endoscopist does not significantly affect diagnostic accuracy[27]. Similarly, our findings were consistent with this. One possible reason for this is that all procedures at our institution are performed under the supervision of an expert.’ Please review the manuscript, line of 340-343.

| Table 3. Prognostic factor for histological diagnostic accuracy. | |  |  |  |  |
| --- | --- | --- | --- | --- | --- |
|  |  |  |  |  |  |
|  | Univariate | |  | Multivariate | |
|  | Crude OR^¶¶^ (95% CI^\|\|\|\|^) | p value |  | Crude OR^¶¶^ (95% CI^\|\|\|\|^) | p value |
| **Age** | 1.00 (0.98–1.03) | 0.656 |  |  |  |
| **Sex, male** | 1.03 (0.59–1.81) | 0.911 |  |  |  |
| **Tumor size** | 1.00 (0.97–1.02) | 0.767 |  |  |  |
| **Expert** | 1.21 (0.64–2.29) | 0.566 |  |  |  |
| **Puncture route, duodenum** | 1.07 (0.53–0.77) | 0.122 |  | 1.17 (0.83-1.65) | 0.379 |
| **Number of punctures** | 1.47 (1.14–1.88) | 0.003 |  | 1.35 (1.04–1.75) | 0.022 |
| **Needle, Franseen** | 2.29 (1.21–4.35) | 0.011 |  | 1.85 (0.95–3.61) | 0.069 |
| **Cut-off value, AUC (95% CI)** |  | | | | |
| Number of punctures | 3, 61.9 (0.54 - 0.69) | | | | |
| Tumor size, (mm) | 29, 51.0 (0.44 - 0.58) | | | | |
| ¶¶: OR, odd ratio, \|\|\|\|: CI, confidence interval | | | | | |
|  | | | | | |

27.	Ishigaki K, Nakai Y, Endo G, Kurihara K, Ishida K, Tange S, et al. Feasibility of comprehensive genomic profiling using endoscopic ultrasound-guided tissue acquisition with a 22-gauge Franseen needle. DEN Open. 2024;4(1):e365.
